# Fucoidan Extracted from *Undaria pinnatifida*: Source for Nutraceuticals/Functional Foods

**DOI:** 10.3390/md16090321

**Published:** 2018-09-09

**Authors:** Yu Zhao, Yizhou Zheng, Jie Wang, Shuyi Ma, Yiming Yu, William Lindsey White, Shiping Yang, Fan Yang, Jun Lu

**Affiliations:** 1 Life and Environment Science College, Shanghai Normal University, 100 Guilin Road, Shanghai 200234, China; 1000441512@smail.shnu.edu.cn (Y.Z.); granger2013@163.com (J.W.); 1000441341@smail.shnu.edu.cn (S.M.); 1000441506@smail.shnu.edu.cn (Y.Y.); shipingy@shnu.edu.cn (S.Y.); bayer@shnu.edu.cn (F.Y.); 2 School of Science, Faculty of Health and Environmental Sciences, Auckland University of Technology, Auckland 1010, New Zealand; lindsey.white@aut.ac.nz; 3 State Key Laboratory of Bioreactor Engineering, East China University of Science and Technology, 130 Meilong Road, Shanghai 200237, China; 4 School of Interprofessional Health Studies, Faculty of Health and Environmental Sciences, Auckland University of Technology, Auckland 1010, New Zealand; 5 Institute for Biomedical Technology, Auckland University of Technology, Auckland 1010, New Zealand; 6 College of Life and Marine Sciences, Shenzhen University, Shenzhen 518060, China; 7 College of Food Engineering and Nutrition Sciences, Shaanxi Normal University, Xi’an 710119, China

**Keywords:** fucoidan, chemical composition, molecular weight, locations, fucose, sulphate, nutraceutical

## Abstract

The importance of fucoidan as a functional ingredient in food, health products, and pharmaceutics is well-recognized due to its beneficial biological effects. Fucoidan is usually extracted from brown seaweeds, including *Undaria pinnatifida*. Fucoidan exhibits beneficial bio-activity and has antioxidant, anticancer, and anticoagulant properties. This review focuses on the biological activity of *U. pinnatifida*-derived fucoidan and investigates its structure–activity or fraction–activity relationship. It also describes several fucoidan extracts, along with their claimed anticancer effects. It aims to provide information and thoughts for future research such as the development of fucoidan into functional foods or nutraceuticals.

## 1. Introduction

**Figure 1:**
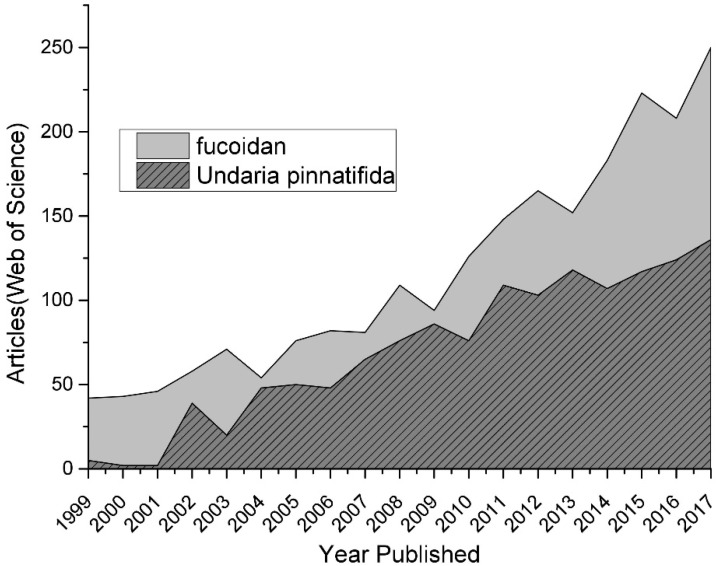
The trend of research on fucoidan as depicted by the number of published articles (Web of Science) in the last two decades. The number of articles was obtained according to topics being assigned in the ISI Web of Science search engine with the following topic search terms: Fucoidan; *Undaria pinnatifida*.

Many studies have focused on marine products, including seaweeds, in the pursuit of novel drugs discovery/development [1]. Fucoidan extracted from *Undaria pinnatifida* is one of these, as it has proven its bioactivity, including anticancer, antioxidant, antiviral, and anticoagulant activities. Fucoidan has garnered increased research interest in both pharmaceutical and functional food industries in recent years (Figure 1).

Fucoidan is a natural sulfated polysaccharide that exists mainly in the cell wall matrix of various species of brown seaweed that are consumed by humans, such as mozuku, kombu, limumoui, bladderwrack, and wakame (the Japanese name of *U. pinnatifida*) [2,3]. It was first isolated by Kylin in 1913 [4], and since then, fucoidan from many different species of brown seaweed has been successfully isolated, each with slightly different structures and biochemical properties. The brown seaweeds containing fucoidan are widely consumed as part of the normal diet in East Asia, particularly in Japan, China, and Korea. Wakame blades are green when cooked and have a subtly sweet flavor and satiny texture. The blades are normally cut into small pieces as they tend to expand during cooking.

In Japan and Europe, wakame is consumed either dried or salted. It is mainly used in soups (particularly miso soup) and salads (tofu salad), or simply used as a side dish. These dishes are typically dressed with soy sauce and vinegar/rice vinegar. In addition, Goma wakame, also known as seaweed salad, is a popular side dish at American and European sushi restaurants. Literally translated, it means “sesame seaweed”, as sesame seeds and oil are usually included in the recipe.

**Figure 2:**
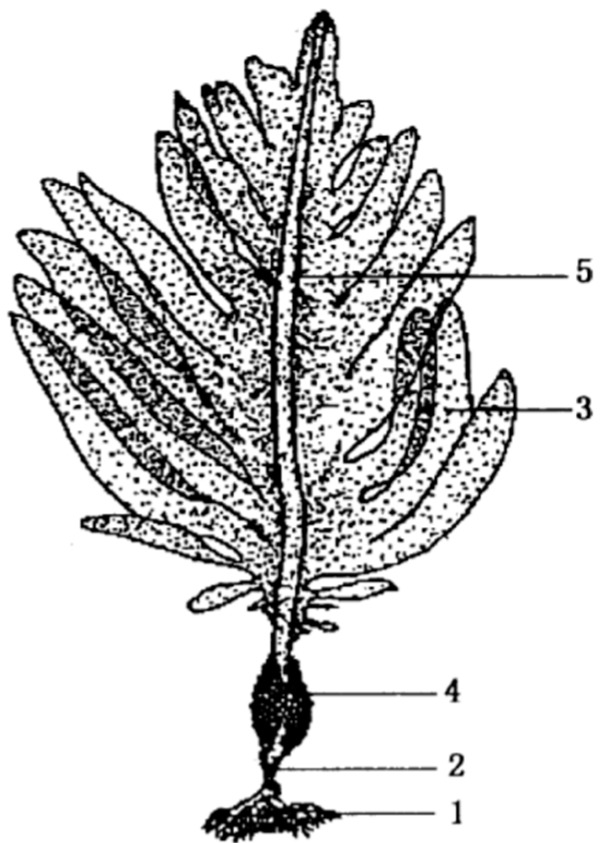
Parts of *Undaria pinnatifida*. 1 = Holdfast, 2 = Stipe, 3 = Blade, 4 = Sporophyll, 5 = Midrib.

**Figure 3:**
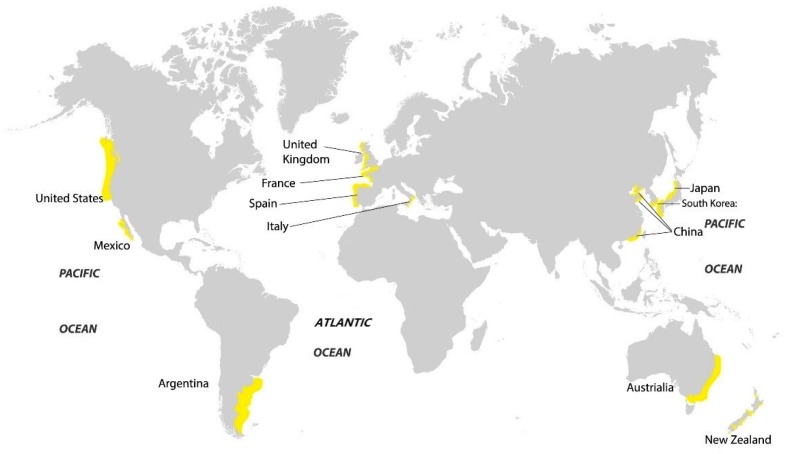
Distribution of *Undaria pinnatifida* around the world.

The brown seaweed species *U. pinnatifida* (Figure 2) is native to the cold temperate seas of China, Japan, and Korea, and has been introduced in many other places including the Europe Atlantic, French Mediterranean, Australia, and New Zealand (Figure 3). It is regarded as a highly invasive species with a high tolerance for light, temperature, and salinity [5,6]. It is also highly fertile with high growth rate and large reproductive output, releasing spores all year round [6,7]. It is farmed extensively in Japan, Korea, and Japan and as such, it is an abundant source from which fucoidan could be extracted and used.

Fucoidan is usually extracted from the sporophyll of *U. pinnatifida* (Figure 2) [8]. However, a key difference between fucoidan from *U. pinnatifida* and those from of other brown seaweed species such as *Fucus vesiculosus* lies in the composition of monosaccharides that form the backbone of the polysaccharide molecule [2,3]. Fucoidan from *U. pinnatifida* is sulfated galactofucan [4]. In contrast, fucoidan isolated from the vast majority of other brown seaweeds mainly consists of sulfated fucose [4]. Literature shows that sulfate content, monosaccharide composition, and structural conformation of fucoidan affect its biological activity [9,10,11]. As such, it is suggested that fucoidan from *U. pinnatifida* with different monosaccharide composition and structural conformation, would possess a wide range of biological activities, which offers itself as an attractive functional ingredient of health products [2,3].

## 2. The Extraction of Fucoidan from *U. pinnatifida*

In 1913, for the first time, Kylin named the polysaccharide extracted from the brown seaweed palmate kelp as fucoidin [12] and it has now been uniformly named fucoidan or sulfated fucan. Fucoidan is a water-soluble polysaccharide. There are a number of commonly used methods to extract fucoidan, including water extraction, acid extraction, microwave-assisted extraction, ultrasonication extraction, and enzyme-assisted extraction [13]. There are reports that the yield of fucoidan varies with different extraction methods [14]. The extraction methods of fucoidan are briefly summarized in Table 1.

## 3. The Structure and Function of Fucoidan from *U. pinnatifida*

In this part, the structure–activity relationship is described. Three clear biological activities: anticancer, antioxidant, and anticoagulant activity are described in detail. The fucoidan structure factors effecting anticancer activity are mainly the content of sulfate and the linking mode of the polysaccharide skeleton, and the main factors affecting the antioxidant and anticoagulant activity are the content of sulfate and the molecular weight of the polysaccharide.

### 3.1. Fucoidan Basic Structure

In 2006, researchers identified the monosaccharides that make up *U. pinnatifida*-derived fucoidan are fucose, xylose, galactose, glucose, rhamnose, and mannose [20]. The three most abundant monosaccharides are mannose, fucose, and galactose [21]. In addition to those basic monosaccharides, uronic acid, and sulfate are also present.

Neutral sugar analysis confirmed that fucose (50.9% mol) and galactose (44.6% mol) are the major neutral sugars of obtained polysaccharide, with small amounts of xylose and mannose. The ratio between fucose and galactose is approximately 1.1:1.0.

**Figure 4:**
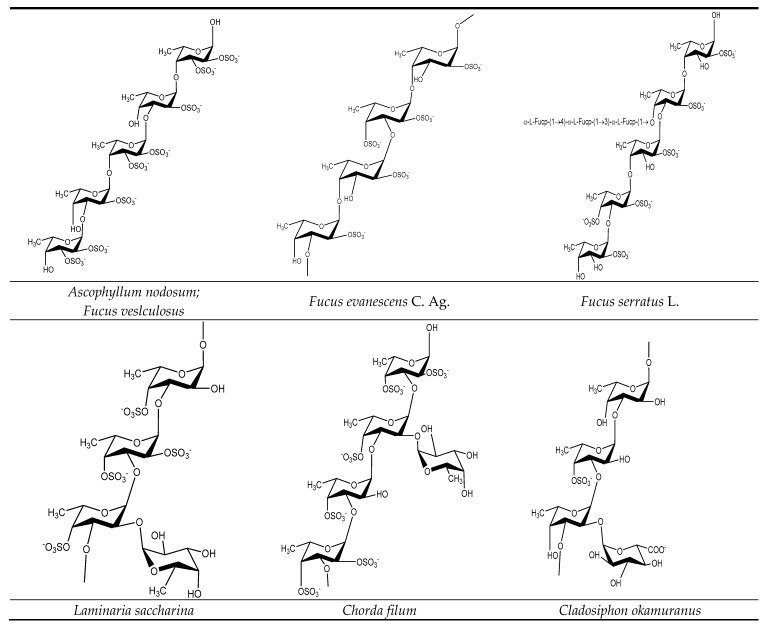
Taxonomic comparison of fucoidan or fucose-containing sulfated polysaccharides structures.

Using analytical methods, including HPLC, gel electrophoresis, elemental analysis, infrared, Raman, and mass spectrometry, it has been found that the connections between fucoidan monosaccharides are 1-3, 1-4, 1-6 glycosidic bonds [16,22]. The various saccharide bonds include 1-3 linked fucose, and 1-3, 1-4, and 1-6 linked galactose. The sulfate substitution sites are mainly at 2- or 4-positions of fucose residues, 3- or 6-positions of galactose residues [22,23]. There is still controversy about the composition and connection of fucose. One claim is that fucose and galactose in galactofucan may form separate blocks and intersperse in one polymer backbone [23]. Another is that there could be two separate polymers: fucan and galactofucan. From ESI-FTICR mass spectrometry analysis, it has been shown that galactose and fucose residues are successively linked to one polysaccharide molecule [23]. There are many kinds of fucoidans that varies with the type of algae extracted. Figure 4 lists the structure of fucoidans extracted from Fucales, Laminariales, and other brown seaweed.

The molecular size of fucoidan is also a significant part of the structural character. Sakai et al. (2003) reported that the molecular weight of fucoidan is 2000 kDa [11]. Fucoidan isolated from the sporophylls of *U. pinnatifida* collected from Wando, South Korea, has an average molecular weight of 2100 kDa [16]. The fucoidan from sporophylls of the same species harvested from a seaweed farm in the coastal area of Kijang, Yangshan County, South Korea, had an average molecular weight of 38 kDa [24,25]. Fucoidans with low molecular weights of 89, 35, 17, and 6 kDa were obtained by radiation-degradation of a 378 kDa fucoidan isolated from *U. pinnatifida* by Park and coworkers [3,26]. It is believed that the difference in molecular weight is caused by different extraction methods [24]. Many studies have shown that the molecular weight of the fucoidan is related to its biological activity.

Sulfate is a characteristic group of fucoidan structure. The content of sulfate is based on the elemental analysis (*DS*, moles of SO_3_ per mol of saccharide units),

(1) $$DS=\frac{\%S}{\%C}(6+2DAc)\frac{A_{C}}{A_{S}}$$

where %S and %C are the carbon and sulfur contents, *A*_C_ and *A*_S_ are the atomic weights of these elements, six and two are the amounts of carbon in the pyran ring and *O*-acetyl group, and *DAc* is the degree of acetylation calculated by 1H NMR.

The results of the elemental analysis show that no protein (non-nitrogen) was detected, while the sulfur content of 9.18% indicated a sulfated polysaccharide. The carbon content is relatively low, which is 23.03% [27]. Infrared spectrum analysis (IR) at 836 cm^−1^ and Raman spectrum analysis at 839 cm^−1^ indicate that the COS bond in the sulfate has bending vibration at the axial C-4 position [28,29].

Igarashi et al. hydrolyzed the sporophyll of Japanese wakame, and the resulting infrared spectrum showed a band at 829 cm^−1^ and it was found that the bending vibration of C–O–S appeared at the C-2 position [15]. There are two other studies show that the bending vibration of C–O–S is present at different carbon locations, thereby indicating that sulfate is a characteristic group of the structure of fucoidan [12,14].

At the same time, it has been confirmed that the sulfate content effected the bioactivity of the fucoidan. Fucoidans extracted from different algae or different locations have different biological activities because it is a polymer mixture. The following Table 2 is a brief summary of the sulfate content detected in fucoidan grown in different countries:

In recent years, individual monosaccharide composition has been found to vary with season. The structural characteristics of individual monosaccharides vary with the collection time [31] and the age of the plant. From April to July in the Northern Hemisphere, fucoidan production increased five-fold. In addition, the monosaccharides constituting fucoidan are significantly affected by the season. The galactose content increases from April’s 20.5% to June/July’s 38–39.5%, and the content of mannose drops. The change in fucose content from April to July was not significant. The ratio of other monosaccharides also changed. The molar ratio of fucose to aspartic acid galactose changes from 1:0.34 to 1:0.66−0.69 [21]. Similar observations are reported by Honya et al. [32] and Fletcher et al. [33]. A New Zealand study also found that sulfate content in the extracted fucoidan increased more than twice within a 4-month period, while fucose content remains constant [34].

### 3.2. Structure Characterization and Structure–Activity Relationship

#### 3.2.1. Anticancer Properties of Fucoidan from *U. pinnatifida*

A recent report shows that fucoidan isolated from the sporophyll of New Zealand *U. pinnatifida* exhibits similar cell growth-inhibition effects in breast adenocarcinoma cell line MCF-7, lung carcinoma cell line A-549, and colon adenocarcinoma cell line WiDr, in comparison with commercial fucoidan (from Sigma, St. Louis, MO, USA) isolated from *F. vesiculosus* [35]). Similar results are reported by another group where breast cancer cell line T-47D and melanoma cancer cell line SK-MEL-28 are susceptible to the anticancer effect of fucoidan isolated from *U. pinnatifida* grown in Japan Sea [2]. Park et al. showed that there was an enhanced inhibitory effect against melanin biosynthesis in B16BL6 melanoma cells with low molecular weight fucoidan [26]. It has also been shown that fucoidan from *U. pinnatifida* has antiproliferation effect on prostate and hepatocellular cancer cells. Research suggests that fucoidan treatment could induce intrinsic and extrinsic apoptosis pathways via the activation of extracellular signal-regulated kinase mitogen-activated protein kinase (ERK1/2 MAPK), the inactivation of p38 MAPK and phosphatidylinositol 3-kinase (PI3K)/Akt signaling pathways, and the downregulation of the Wnt/β-catenin signaling pathway [36]. Further research suggested that fucoidan induces apoptosis via a ROS-mediated mitochondrial pathway. By increasing reactive oxygen species (ROS) production, fucoidan induces mitochondrial oxidative damage, mitochondrial membrane potential (MMP) depolarization, and release of cytochrome c; combined with downregulation of Livin and XIAP mRNA and activation of caspase-3 and caspase-9 [37]. Another report demonstrates that fucoidan can ameliorate hepatic infrared injury in mice via JAK2/STAT1-mediated apoptosis and autophagy [38].

The anticancer activity of fucoidan is influenced by its sulfate content; low molecular weight fucans isolated from *Ascophyllum nodosum* exhibited increased antiproliferative activity on fibroblast cell line CCL39 with increased sulfate content [33]. Likewise, oversulfated fucoidan from *F. vesiculosus* exhibited higher anti-angiogenesis potency on the growth of B16 melanoma cells, Lewis lung carcinoma, and Sarcoma 180 cell lines [34]. This suggests that the sulfate content of fucoidan may be critical in influencing its anticancer activity.

More interestingly, fucoidan isolated from *U. pinnatifida* exhibits stronger anticancer activity against breast cancer T-47D and melanoma SK-MEL-28 cell lines as compared to fucoidan isolated from *Saccharina japonica*. These fucoidans induced apoptosis in relevant cancer cell lines as well as they have antimetastatic activity blocking the interactions between cancer cells and the basement membrane. The authors attribute the higher anticancer activity to the types of glycosidic bonds that it possesses, where *U. pinnatifida* fucoidan has a backbone structure of (1→3):(1→4)-*O*-glycosidic bonds and *S. japonica* fucoidan has a backbone structure of (1→3)-*O*-glycosidic bonds [2]. Fucoidan has also shown considerable anticancer potential against human liver cancer (HepG2) cells (LD 50, 18.01 ± 1.2 μg/mL) . As such, it can be concluded that both sulfate content and types of glycosidic bonds play important role in determining the anticancer activity of fucoidan.

#### 3.2.2. Antioxidant Activities of Fucoidan from *U. pinnatifida*

The antioxidant capacity of fucoidan isolated from various seaweed species has been demonstrated in the literature . It has been reported that fucoidan typically exhibits strong secondary antioxidant activity that is comparable to synthetic antioxidants such as butylated hydroxyanisole (BHA) and butylated hydroxytoluene (BHT) that are known for causing side effects in humans including cancer [31,39]. It has been reported that fucoidan isolated from *Sargassum binderi* exhibits significantly higher secondary antioxidant capacity, based on superoxide radical scavenging and hydrogen peroxide scavenging assays, than synthetic antioxidants BHA and BHT [38]. In another study, it was concluded that fucoidan isolated from *Laminaria japonica* exhibited significantly higher superoxide radical scavenging activities as compared to BHA, BHT, and α-tocopherol [40]. This highlighted the potential of fucoidan as a source of antioxidant. Fucoidans extracted from *U. pinnatifida* from mussel farms in New Zealand, exhibit strong antioxidant activities using the DPPH scavenging and CUPRAC assays [34]. Another recent study prospects fucoidan from *U. pinnatifida* as a potential antioxidant that can effectively abrogate oxidative stress, too [41].

There have been numerous reports on the correlation between the antioxidant capacity of fucoidan and its sulfate content and molecular weight. In the same study conducted by Wang et al., it reports that the superoxide radical scavenging activity of the fucoidan sample enhances with increased sulfate content [40,42]. In a separate study, a similar trend has been observed where fucoidan extracted from *F. vesiculosus* and *Padina gymnospora* exhibit significantly higher antioxidant activity than fucan fractions with lower sulfate content [43,44,45].

Besides sulfate content, a correlation between molecular weight and the antioxidant capacity of fucoidan has also been reported in the literature [4]. One study compares the antioxidant capacity of five fucoidan fractions, with different molecular weight and sulfate content, isolated from *L. japonica* [46]. It shows that the high molecular weight fucoidan fractions show low inhibitory effects on low-density lipoprotein (LDL) oxidation while the low molecular weight fractions exhibited higher inhibitory effects [47]. It is also worth noting that all fractions contained different sulfate contents. Hence, this suggests that both sulfate content and molecular weight are important determinants of the antioxidant activity of fucoidan.

#### 3.2.3. Anticoagulant Activity of Fucoidan from *U. pinnatifida*

Previous studies have confirmed the anticoagulant and antithrombotic activity of fucoidan from the brown seaweeds *Saccharina latissimi* [48]. According to previous reports, the molecular weight of the fucoidan polymer is related to its anticoagulant activity [49]. One study found that the fucoidan polymer exhibited the strongest anticoagulant activity with the molecular weight from approximately 10 kDa to 300 kDa [9]. The activity of fucoidan extracted from *U. pinnatifida* on red blood cells has been studied by Caterina Faggio et al. Results show that fucoidans appeared to have no cytotoxic effect on the red blood cells, and the values of prothrombin time, activated partial thromboplastin time, and fibrinogen are significantly changed. The purified fucoidan significantly prolongs clotting time [50,51]. Various natural anticoagulants moderate the secondary hemostasis pathway and currently, heparin or low-molecular-weight heparin (LMWH) is frequently used clinically [52,53]. The current source of heparin is mainly from the animals, including from pig intestine or bovine lung. It is a commonly used natural medicine. The fucoidans interfere with both the extrinsic and intrinsic pathways of coagulation inhibiting clot formation, which has an action similar to that carried out by heparin. The development of anticoagulant drugs with fucoidans would be advantageous since their use would avoid the potential for contamination with prions or viruses in commercial heparins that are obtained from pig and bovine intestine. Moreover, with more specific activities or targets, fucoidans could find applications complementary to heparin. Therefore, new researches on discovering novel anticoagulants are still favored [54], and the above researches suggest that fucoidan from *U. pinnatifida* is a potential useful source of anticoagulant drug.

#### 3.2.4. Antibacterial Activity

There have been a large number of reports on compounds derived from algae with antibacterial activity, such as acrylic acid, halogenated aliphatic compounds, terpenes, sulfur-containing heterocyclic compounds, and phenolic compounds [55,56]. However, few studies have been found in the literature regarding *U. pinnatifida*-derived fucoidan with antibacterial activity.

**Figure 5:**
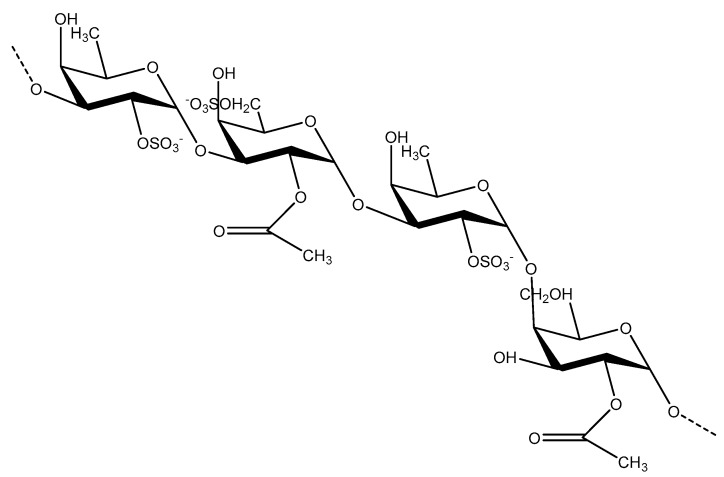
Basic structure of fucoidan from *Undaria pinnatifida.*

The antibacterial mechanism is due to a large amount of sulfuric acid and glucuronic acid in the depolymerization products of fucoidan, which have the property of polyanion. The depolymerized fucoidans bind to the bacterial membrane proteins and cause a membrane-disrupting effect that induces the expression of certain apoptotic factors, which leads to bacterial apoptosis. Antibacterial activity of fucoidan from *U. pinnatifida* has been tested and proven to be effective. The results show that the maximum zone of inhibition of the bacterial growth against *S. aureus* is 15.67 ± 0.76 mm. Compared with Gram-negative strains, Gram-positive bacterial strains are more inhibited by fucoidan. It may be due to the inhibition of peptidoglycan formation, or the presence of special cell wall components of Gram-negative bacteria that act as a barrier for fucoidan. The molecular mechanism of the activity needs to be further studied. Although, fucoidan has complex structure and the effective structure is not clear, the structural backbone has been elucidated by a recent study. The basic structure of fucoidan from *U. pinnatifida* is presented in Figure 5.

Based on these results, it can be concluded that fucoidan as a natural product can be used as a lead for the development of new drugs with various therapeutic potentials. Table 3 lists the biological functions of fucoidan extracted from *U. pinnatifida*. In addition to the above activities, fucoidan has other functions, such as anti-allergy. An experiment by Jiao and coworkers suggests that fucoidan might ameliorate allergic reactions [57].

The literature shows that the production of well characterized and reproducible fucoidan fractions on a commercial scale is possible. Thus, therapies from fucoidan could become a realizable goal [58]. Current status in fucoidan drug discovery, mechanisms of action and role of the well-defined structures has been summarized [59]. Accurate structure–function relationships have mostly been achieved when sulfated fucans and sulfated galactans of well-defined structures are used. These types of glycans become vital to identify the fucoidan chemical composition needed to achieve satisfactory clinical responses [59].

## 4. Fucoidan as Functional Food and Therapeutic Agent

Functional food is foods that have been demonstrated to provide specific health benefits beyond the basic nutrition. The design of functional foods is hence associated with the concept of preventing diseases and/or improving the health of consumers, besides the basic nutrient needs. Notably, fucoidans have been evidenced to play a vital role in human nutrition and health on the account of their biological activities and health benefits. Fucoidan from *U. pinnatifida* possesses great potential to be used as a functional food to reduce diseases or as a supplement for alternative therapy.

Quite a number of functional foods using *U. pinnatifida* containing fucoidan have been developed, and related patents have been registered. Table 4 lists some of those products (cookie, beverages, noodles, tea, and restructured meat) and their claimed benefits. Furthermore, fucoidan based therapies have been shown to have reached various beneficial therapeutic goals in arthritis, blood homeostasis, influenza, and malaria (Table 5). Apart from the above-mentioned therapeutic potentials, fucoidan is also able to mediate cell differentiation and exert anti-inflammatory activity [60].

In a recent randomized, double-blind, controlled clinical trial, low-molecular-weight fucoidan has been used as a supplement to chemotherapeutic agents in patients with metastatic colorectal cancer (mCRC). It has been shown that the disease control rate has been significantly improved with this supplement, in a median follow-up period of 11.5 months. It is noteworthy that this is the first clinical trial evaluating the efficacy of fucoidan fraction as a supplemental therapy in the management of patients with mCRC [61].

To classify fucoidan as a supplemental therapeutic agent requires the possible interaction between fucoidan and other therapeutic agents to be studied. A recent report shows the effect of co-administration of fucoidan, derived from *U. pinnatifida*, on the pharmacokinetics of two commonly used hormonal drugs, letrozole and tamoxifen, in patients with breast cancer. Results suggest that fucoidan in the studied form and dosage could be taken concomitantly with letrozole and tamoxifen without the risk of significant pharmacokinetic interactions [62].

## 5. Conclusions

**Figure 6:**
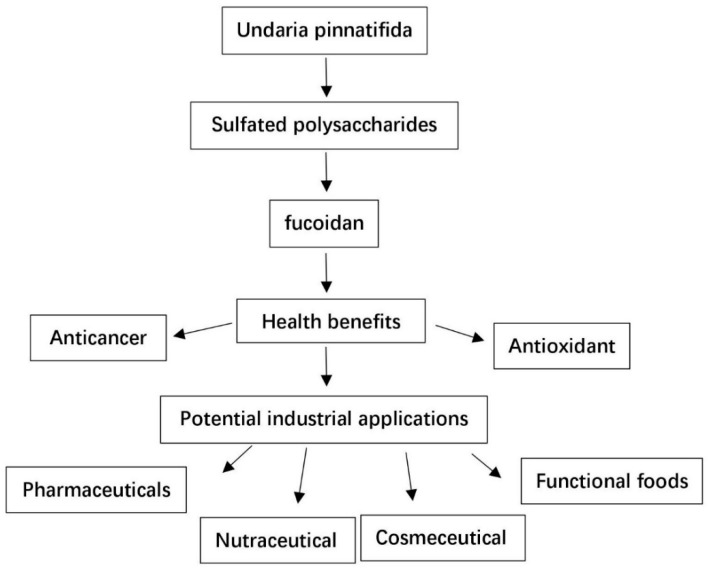
Biological properties and potential industrial uses of *Undaria pinnatifida*-derived fucoidan.

Over the recent years, there have been visible developments in the fields of functional food, nutraceutical, cosmeceutical, and pharmaceutical. There is a growing demand from the public to get food products that provide health benefits instead of just basic nutrition. There is an increasing awareness among consumers about health-promoting foods worldwide. Therefore, many new functional food products have been developed and are being developed with natural ingredients that provide health benefits. As reviewed above, there are extensive scientific evidence that *U. pinnatifida*-derived fucoidan possesses various health benefits. This opens up the potential of developing products containing *U. pinnatifida*-derived fucoidan into functional foods, nutraceuticals, cosmeceuticals, and even drugs (Figure 6). However, it is important to study how to retain and/or improve the functional properties (bioactivities) of fucoidan in different industrial processes, for example, increasing low molecular weight fucoidan percentage and/or the degree of sulfation. Another important consideration is the consumer’s acceptance of the sensory properties of the newly developed fucoidan-containing products. Furthermore, developing new and advanced processing technologies will ensure the exploitation of fucoidan’s beneficial bioactivities from those newly developed products.

## Figures and Tables

**Table 1:** Extraction methods and their rationale, properties, and the yield in each best condition.

Extraction Method	Rationale	Properties	Extraction Conditions	Yield	Reference
Hot water extraction	A method based on the solubility of fucoidan in hot water and the insolubility in ethanol and other organic solvents.	Low cost, simple operation, but time-consuming and using a large amount of solvent.	*U. pinnatifida* was washed by ethanol at 80 °C for 1 h, then was extracted in 60-time weight of water for 7 h at 100 °C. Supernatant was precipitated with ethanol.	12.9%	Jia et al. [15]
Dilute acid extraction	Based on the solubility of fucoidan in dilute hydrochloric acid aqueous solution. It is difficult for fucoidan to dissolve at lower pH value but easy for part of its sodium salt.	The extraction rate is not high, and the structure of fucoidan is easy to be destroyed which affects the bioactivity.	250 g dried sporophyll was added to 4 L of 0.1 N HCl for 24 h at ambient temperature. The extract was filtered, and the filtrate was neutralized with 1 N NaOH. Fucoidan was precipitated with 3:1 volume of 75% ethanol.	3.9%	Kim et al. [16]
Microwave-assisted extraction	Microwave radiation penetration has high energy, which can shortening extraction time to improve efficiency and reduces the use of organic solvent.	Fucoidan is extracted more selectively and quicker with better yields, using less energy and solvent, reducing costs and waste, and less destructive to the structure	1 g milled dry seaweed was suspended in 25 mL of distilled water and placed into the extraction vessel. The suspensions were irradiated under 120 psi pressure for 1 min.	18.2%	Rodriguez-Jasso et al. [17]
Ultra-sonication extraction	Ultrasonication produces cavitation, directs dynamic shock waves on the surface of materials. It breaks the cell wall of organic materials and facilitate the extraction of fucoidan.	Higher extraction yields and lower damage to fucoidan structure	10 g powdered materials were added to 0.1 N HCl (PH = 2) and treated by ultrasonication at room temperature, 80% amplitude for 6 h. The supernatant was neutralized (PH = 7) with 0.1 N NaOH.	33.0 ± 0.4%	Song et al. [18]
Ultra-filtration membrane extraction	It is the use of enzymes to disrupt the structure of cell walls, promote the dissolution of fucoidan, and greatly shorten the extraction time.	Extract fucoidan effectively while maintaining its structure and biological activity	Algae powder:water at 1:20 ratio was pressed at 25 °C to become seaweed slurry. Slurry at PH = 6.0 was added 2.0% enzyme and reacted for 2 h at 40 °C, then heated to 80 °C rapidly and extracted for 1 h, centrifuged, dialyzed, precipitated by ethanol, and finally dried.	7.76%	Wang et al. [19]

**Table 2:** The mass fraction of fucoidan derived from *U. pinnatifida* grown in different countries.

Region	Process	Mass Fraction	Ref.
China	Extracted and purified polysaccharide from Wakame	43.20% polysaccharide, 12.70% sulfate, 9.78% glucuronic acid	[2]
South Korea	Purified fucoidan by HPLC	52.34% neutral sugar, 26.2% uronic acid, 7.4% sulfate ester	[16]
New Zealand	*U. pinnatifida* was harvested from Port Underwood, New Zealand	Monosaccharide composition: fucose (39.24%), xylose (28.85%), galactose (26.48%), mannose (5.04%), glucose (0.95%). The minor components: sulfate (15.02%), uronic acid (1.24%), and protein (0.36%).	[30]

**Table 3:** Biological functions of *U. pinnatifida.*

Function	Origin	Effective Molecule/Fraction	Mode of Action	Ref.
Anti-lung carcinoma	Japan	Low molecular weight fucoidan fraction (5–30 kDa)	Induce apoptotic damage to A-549 cell lines	[35]
Anti-colon adenocarcinoma	Japan	Low molecular weight fucoidan fraction (5–30 kDa)	Induce apoptotic damage to WiDr cell lines.	[35]
Anti-breast cancer	Japan	A partially acetylated galactofucan with a high degree of sulfation and its main chain is built up of (1→3)- and/or (1→4)-a-l-fucopyranose residues.	Induce apoptosis in cancer cell lines as well as having antimetastatic activity blocking the interactions between cancer cells and the basement membrane.	[2]
Anti-melanoma	Japan	Same as the above.	Same as the above.	[2]
	Korea	Fucoidan with low molecular weights of 89, 35, 17, and 6 kDa (prepared by radiation-degradation of a 378 kDa fucoidan)	Fucoidan inhibits tyrosinase and increases radical scavenging activity.	[26]
Antioxidant	Korea	Fucoidan with high sulfate content	Fucoidan directly scavenges the free radicals produced inside the body, effectively abrogate oxidative stress.	[47]
Anticoagulant	Italy	Sulfonated polysaccharides of fucoidan	The sulfonated polysaccharides interfere with both the extrinsic and intrinsic pathways of coagulation inhibiting clot formation, that has an action similar to that carried out by heparin.	[51]
Antibacterial	Korea	Sulphated polysaccharides of fucoidan	Inhibition of peptidoglycan formation, or the presence of special cell wall components of Gram-negative bacteria that act as a barrier for fucoidan.	[55]

**Table 4:** Several products formulated with the use of *U. pinnatifida* and/or extracts.

Functional Food	Processes/Preparation	Related Parameters	Food Sensory Evaluation	Results	Ref.
Cookie	Raw materials were mixed with water to form a dough piece with 2 mm thick, finally baked	Wakame powder 20%	Crisp taste, delicious flavor	Have the effect of reducing weight and blood glucose.	[63]
Beverages	Raw juice was obtained after *U. pinnatifida* was fermented by 8% inoculated yeast at 30 °C for 24 h.	50% raw wakame juice.	Rich aroma of wakame and fermentation, sweet and sour taste, no smell and other odor.	Lowering blood pressure, lipids, and cholesterols, impeding platelet aggregation and preventing arteriosclerosis.	[64]
Noodles	Wakame, wheat flour and wheat gluten flour were used.	No details	Yellow-green color, slight fragrance of wakame.	Suitable for patients with high blood pressure, glucose and lipids	[65]
CVDs-related parameters in foods using *U. pinnatifida*					
Tea	Wakame was dried with hot air, crushed to pieces, boiled with water, and filtrated.	ACE inhibition IC_50_: 26.4 ± 1.05 mg/mL.	Green color	Contains rich minerals and suppresses hypertensive activity of angiotensin I.	[66]
Restructured meat	Dried wakame was homogenized with raw meat.	*U. pinnatifida* at 5%	No details	Moderately ameliorated lipid profile in hypercholesterolemic rats.	[67]
Gel/emulsion meat systems	No details	*U. pinnatifida* at 5.6%	No details	Increase *n*-3 PUFA and antioxidant, Decrease *n*-6/*n*-3 PUFA ratio and sodium.	[68]

CVDs—cardiovascular diseases; ACE—angiotensin I converting enzyme; PUFA—polyunsaturated fatty acids.

**Table 5:** Fucoidan based therapies and related parameters.

Therapeutic Items	Trial	Administration	Observation	Ref.
Arthritis	Collagen-induced arthritis in mice	100 kDa and 1 kDa fractions orally administered daily 300 mg/kg for 49 days	1 kDa fraction effectively inhibited whereas 100 kDa exacerbated disease	[69]
Blood Homeostasis	Healthy human subjects	3 g of *U. pinnatifida* fucoidan were ingested daily for 12 days by healthy subjects	A significant prolongation of global clotting time was noted	[70]
Influenza	In vivo	Orally delivered less than 1 mcg/mL	A marked inhibitory effect on the recent H1N1 *Influenza A* virus	[71]
	Mouse model	5 mg per day orally delivered	Strongly inhibited *Influenza A* infection	[72]
Malaria	*Plasmodium berghei*-infected mice	Orally delivered for 4 days	A 37% suppressive effect and a significant delay in the deaths from anemia	[73]
